# Work Hard… Drink Hard? Occupational, Sociodemographic and Health Determinants of High‐Risk Alcohol Consumption Among Australian Workers

**DOI:** 10.1111/dar.70092

**Published:** 2025-12-16

**Authors:** Gianluca Di Censo, Kirrilly Thompson, Jacqueline Bowden

**Affiliations:** ^1^ National Centre for Education and Training on Addiction, Flinders University Adelaide Australia; ^2^ College of Medicine and Public Health, Flinders Health and Medical Research Institute, Flinders University Adelaide Australia; ^3^ School of Medicine and Public Health, University of Newcastle Newcastle Australia

**Keywords:** alcohol‐related harm, behavioural health, employee well‐being, occupational determinants of health, risk factors, workforce epidemiology, work‐related stress

## Abstract

**Introduction:**

Although occupational stressors are acknowledged as contributors to high‐risk alcohol consumption, their impact relative to sociodemographic and health factors is not well understood. This study investigated how occupational stressors compare with sociodemographic and health factors in influencing three high‐risk drinking patterns among Australian workers.

**Methods:**

This analysis included 26,255 employed individuals (48.2% female) drawn from 23 waves (2001–2023) of the Household Income and Labour Dynamics in Australia survey. The study conducted multivariate regressions on three types of high‐risk drinking: (i) high‐risk drinking across the week; (ii) single‐occasion high‐risk drinking; and (iii) any high‐risk drinking.

**Results:**

Working more than full‐time hours was associated with higher odds of high‐risk drinking across the week (OR 1.12, 95% CI [1.05, 1.19]). Shift workers were associated with single‐occasion high‐risk drinking (OR 1.09, 95% CI [1.02, 1.16]). Desiring to work more hours was associated with single‐occasion high‐risk drinking (OR 1.16, 95% CI [1.09, 1.24]). Workers aged 18–29 years had a twofold increased odds of any high‐risk drinking (OR 1.99, 95% CI [1.77, 2.23]), while women had decreased odds (OR 0.11, 95% CI [0.10, 0.13]). Smoking was the modifiable risk factor most strongly associated with any high‐risk drinking (OR 2.80, 95% CI [2.60, 3.02]).

**Discussion and Conclusions:**

This analysis found that occupational factors were associated with high‐risk drinking; however, their impact was less substantial than sociodemographic and health factors. Interventions targeting risk factors among high‐prevalence groups and health promotion campaigns highlighting the health risks associated with high‐risk alcohol consumption are warranted.

## Highlights

- Workplace stressors—particularly physical stressors—explain some of the variance in high‐risk drinking patterns in Australian workers.
- Sociodemographic factors (age, sex, education, etc.) explain far more of the variance in high‐risk drinking.
- Smoking was strongly associated with high‐risk drinking patterns, indicating a need for universal interventions aimed at reducing all health risk behaviours.

## 1 Introduction

High‐risk alcohol consumption is associated with a range of adverse health outcomes [1, 2, 3, 4], and it contributes substantially to both the global and Australian burden of disease [5, 6]. In Australia, over one‐third of working‐aged adults consume alcohol at levels that place them at increased risk of harm [7]. Although it is purported that occupational factors (e.g., working hours, job satisfaction, job demands) contribute to alcohol use [8, 9], few studies have examined how much variance they uniquely explain in alcohol consumption. Understanding the extent to which occupational factors explain high‐risk drinking is essential for informing more effective and targeted public health and workplace interventions.

Frone and Bamberger [10] argue that factors external and internal to the workplace play a role in influencing workers' alcohol and other drug use. Factors external to the workplace encompass sociodemographic aspects—such as age, sex, socio‐economic status and income—and health risk behaviours, including substance use habits. For example, evidence indicates that younger individuals face a heightened risk of participating in high‐risk single‐occasion drinking [11, 12] and developing alcohol use disorder [13], whereas older individuals tend to consume alcohol more regularly [14, 15, 16]. Research also indicates that men are more likely to engage in alcohol consumption than women [12, 17, 18], to drink in excess of health guidelines [7] and to have higher rates of alcohol use disorder [13, 19]. Consequently, the current body of research illustrates a strong association between various factors external to the workplace and the consumption of alcohol.

Factors internal to the workplace—or occupational stressors—can also increase the likelihood of high‐risk alcohol consumption [10]. Frone [9] presents a framework suggesting that such stressors arise from two distinct work‐related domains: workplace structural demands and psychosocial work‐related stressors. Workplace structural demands refer to objective, externally imposed features of the work environment, such as work hours, schedules and physical working conditions. Research on shift workers has shown that alcohol use is often reported as a strategy to facilitate sleep [17, 20], despite evidence that alcohol actually impairs sleep quality [21, 22]. Dorrian and Skinner [18] observed that shift workers exhibited a greater propensity for binge drinking, although their total weekly alcohol intake did not significantly exceed that of non‐shift workers. Research on fly‐in/fly‐out workers in the Australian mining sector demonstrates that shift workers are also more prone to binge drinking, particularly during their days off [23]. Similarly, working more than the typical full‐time hours (i.e., > 40 h) has been associated with high‐risk alcohol use [19, 24, 25]. Schluter et al. [15] found a positive correlation between long working hours and alcohol consumption among Australian and New Zealand nurses. Together, these findings suggest that the structural characteristics of working conditions—particularly time‐based demands—can contribute to alcohol‐related harm.

In addition to workplace structural demands, Frone's [9] framework identifies a second domain of occupational stressors: psychosocial work‐related stressors. This refers to the subjective or perceived stressors associated with work [8]. For instance, it has been proposed that job satisfaction may inversely influence workers' alcohol consumption [26]. Similarly, perceived job stress has been associated with high‐risk alcohol consumption in some studies [27, 28, 29], while others have found no such link [15, 30]. Research on job autonomy—defined as an individual's control over decisions at work—has also yielded inconsistent results, with some studies observing a positive association with high‐risk drinking [27, 31, 32] and others reporting no association after adjusting for relevant covariates [33, 34]. In contrast, job insecurity has consistently emerged as one of the few psychosocial work‐related stressors with a robust link to alcohol consumption [34, 35, 36]. In summary, the existing literature presents strong evidence regarding the impact of workplace structural demands on alcohol consumption, while the findings related to psychosocial work‐related stressors are comparatively less robust.

### 1.1 The Present Study

Despite an increased focus on investigating the relationship between occupational factors and alcohol use, little is known about whether occupational variables explain more of the variance in high‐risk drinking among Australian workers than sociodemographic or health factors. Therefore, the present study examines whether occupational factors account for additional variance in three high‐risk drinking patterns among Australian workers, beyond that explained by sociodemographic and health variables. The three patterns of high‐risk drinking are defined as follows: high‐risk drinking across the week (exceeding 10 standard drinks in a week); single‐occasion high‐risk drinking (surpassing four standard drinks in a single occasion); and any high‐risk drinking (exceeding either the across‐the‐week or per‐occasion limits). This aligns with the first principle outlined in Australia's Alcohol Guidelines as established by the National Health and Medical Research Council [37]. The implications of this research are important for informing public health and workplace interventions aimed at reducing high‐risk alcohol consumption by addressing the factors influencing alcohol use at both the workplace and personal levels.

## 2 Methods

### 2.1 Participants

This study used data from the Household, Income and Labour Dynamics in Australia (HILDA) survey, a large‐scale national longitudinal survey. Twenty‐three waves of data were used for the present analysis. The HILDA survey collects data pertaining to several variables, including alcohol consumption, health, work arrangements and psychological and physical well‐being. The methodology of data collection has been described in detail elsewhere [38]. The number of participants who completed the survey for each wave ranged from *n* = 12,408 (Wave 4) to *n* = 17,693 (Wave 16), while response rates ranged from 52.4% (Wave 23) to 86.9% (Wave 2). The total sample size for the dataset was *N* = 47,478 across the 23 time points from 2001 to 2023. For this study, a subset of observations from employed individuals aged 18 years and older was selected, resulting in a final analytical sample of *N* = 26,255 (48.2% female) and *N* = 216,641 observations. This secondary data analysis of de‐identified data was approved by the custodians of the HILDA dataset.

### 2.2 Measures

The outcome variable in this study was high‐risk alcohol consumption, as defined by Guideline 1 of the Australian Alcohol Guidelines [37]. This was operationalised using three binary variables: (i) across‐the‐week high‐risk drinking (> 10 standard drinks per week); (ii) single‐occasion high‐risk drinking (> 4 standard drinks per occasion); Socio‐Economic Indexes for Areas and (iii) any high‐risk drinking (> 10 standard drinks per week or > 4 standard drinks per occasion). An ordinal variable in the HILDA dataset quantified daily alcohol consumption. Responses ranged from ‘1–2 standard drinks’ to ‘13 or more standard drinks’ per drinking occasion. This variable was recoded such that responses of > 4 standard drinks were classified as *single‐occasion high‐risk drinking*. *Across‐the‐week high‐risk drinking* was derived from two variables. The first was alcohol consumption per drinking occasion, which was recoded to approximate continuous values (e.g., ‘1–2 standard drinks’ = 1.5, ‘3–4 standard drinks’ = 3.5). The second variable was typical drinking frequency, with responses ranging from ‘never drunk alcohol’ to ‘daily.’ This variable was recoded to approximate continuous values (e.g., ‘3 or 4 days weekly’ = 3.5, ‘5 or 6 days weekly’ = 5.5). Alcohol consumption per drinking occasion was multiplied by typical drinking frequency to approximate total weekly alcohol intake. Scores of > 10 standard drinks per week were coded as engaging in across‐the‐week high‐risk drinking. Participants were classified as engaging in *any high‐risk drinking* if they were identified as either across‐the‐week or single‐occasion high‐risk drinkers. We excluded abstainers from the analyses, as combining them with individuals who consume alcohol at low or moderate levels could introduce bias [39].

The study examined key variables related to occupation‐related factors that are associated with high‐risk alcohol consumption. Individuals who worked outside ‘normal’ daytime schedules (i.e., those working outside 8 AM to 6 PM) were classified as shift workers [40]. The employment commitment of workers was coded into a categorical variable with three levels: part‐time (< 35 h), full‐time (35–40 h) and more than full‐time (> 40 h). Three psychosocial job adversity scales [41]—job demands and complexity (five items, *α* = 0.73), job control (three items, *α* = 0.83) and job security (four items, *α* = 0.62)—were derived from the HILDA survey. We determined each of the scale scores by calculating their respective means of items on a seven‐point Likert scale (score range = 1–7), which we did to facilitate interpretation (e.g., a one‐point increase in scores on a seven‐point scale was associated with an *x*‐fold change in the odds of the outcome). Job satisfaction was evaluated using a 10‐point scale, which ranged from completely dissatisfied to completely satisfied. For the purposes of this study, scores ≤ 5 were coded as dissatisfied or indifferent, while those > 5 were coded as satisfied. Finally, preference of work hours had three categories: ‘less hours’, ‘same hours’ and ‘more hours’.

The current investigation used various sociodemographic variables as independent variables in the models, which included sex, age, rurality and partnership status. Educational attainment was recoded for ease of interpretation: higher education (i.e., master's, doctorate, graduate diploma or certificate, bachelor's or honours), vocational (i.e., Certificate III or IV, diploma or advanced diploma), Year 12 and Year 11 and below. In addition, the 2021 Socio‐Economic Indexes for Areas was used to assess participants' relative socio‐economic advantage or disadvantage [42], which was subsequently recoded from deciles to quintiles for the analyses conducted in this study. Income was measured using two variables: one capturing positive income values (gains) and the other capturing negative income values (losses). These variables were combined to create a total income figure [43]. Total income was inflation‐adjusted to the 2023 Consumer Price Index rate [44] and subsequently divided into quartiles for the analyses: Q1 (≤ AUS$42,948), Q2 (AUS$42,949–AUS$69,949), Q3 (AUS$69,950–AUS$105,008) and Q4 (> AUS$105,000). Health‐related factors included two binary variables: long‐term health condition and current smoking status. Individuals were classified as smokers if they smoked daily, weekly or less frequently than weekly (but still smoked). Response options for categorical variables can be found in Table 1.

### 2.3 Analytic Strategy

Data analyses were performed using R (version 4.5.1). Three generalised linear mixed effects models (GLMM) with a binomial distribution were fitted using glmmTMB [45] to examine factors associated with high‐risk alcohol consumption (any, across‐the‐week and single‐occasion). GLMMs were selected due to their flexibility in addressing the correlated nature of repeated measures in longitudinal data [46]. GLMMs use likelihood‐based methods—in this case, restricted maximum likelihood estimation—which do not require balanced datasets because they handle missing outcome data well [47] even without multiple imputation [48]. Observations with missing values on model predictor variables were excluded from the relevant analyses (complete case with respect to predictor variables).

The models included random intercepts (participant ID) to account for the repeated measures across participants and random slopes (survey wave) to account for individual differences in change across time. For each of the outcome measures, three models were fitted: a null model, a basic model (including only sociodemographic and health variables) and a full model (encompassing all relevant variables). The models were evaluated using the Akaike information criterion, a method for determining how well the model fit the data, which revealed that the full model fitted the data best for each of the outcome measures. Since the data were unbalanced (i.e., participants did not necessarily complete all the waves), it was unfeasible to apply survey weights; consequently, this analysis should be considered exploratory and not representative of the Australian population.

## 3 Results

### 3.1 Participant Description

The mean age of participants across all waves of data was *M* = 40.3 (SD = 13.6), and 48.2% were female. The sample will be described in terms of observations rather than unique participants due to the data being longitudinal. The largest age group in the sample was 50+ (27.7%), followed by 18–29 (26.6%), 30–39 (22.9%) and 40–49 (22.8%). Most participants across all data waves resided in major cities (64.2%). Regarding socio‐economic status, the largest proportion of the sample belonged to the most advantaged category (23.0%), while those in the most disadvantaged category constituted the smallest percentage of respondents (16.8%). The most common level of educational attainment was vocational (34.0%), followed by higher education (31.0%). Around two‐thirds of the sample were partnered (68.9%). Nearly one‐fifth of the sample were tobacco smokers (19.0%) and 17.1% had a health condition.

The majority of the sample (76.6%) followed a normal work schedule, while the remaining (23.4%) worked shifts. In terms of work commitment, 36.3% of the sample worked full‐time (i.e., 35–40 h/week) and 32.5% worked more than full‐time (i.e., > 40 h/week). The occupation type most represented in the sample was professionals (25.1%), followed by managers (14.3%) and clerical and administrative workers (14.1%). Furthermore, 59.9% of the sample indicated that their working hours were sufficient, while 14.4% expressed a desire to work additional hours. The prevalence of job dissatisfaction within the sample stood at 9.4%. Regarding alcohol consumption, 36.8% of respondents reported engaging in high‐risk alcohol consumption, with 25.8% participating in high‐risk consumption across the week and 22.9% in single‐occasion high‐risk consumption. Table 1 displays the descriptive statistics for key variables grouped by high‐risk alcohol consumption categories (any, across‐the‐week or single‐occasion).

**TABLE 1:** Descriptive statistics of the sample, stratified by high‐risk alcohol consumption (any, across‐the‐week or single‐occasion).

	High‐risk drinking			Total sample	
	Any	Across‐the‐week	Single‐occasion		
	%	%	%	*n*	%
Sex					
Male	47.0	34.9	30.2	112,152	51.8
Female	25.8	16.0	15.1	104,489	48.2
Workforce age					
Junior (18–29 years)	45.3	21.2	39.0	57,527	26.6
Junior‐mid (30–39 years)	34.1	23.2	22.1	49,589	22.9
Mid‐senior (40–49 years)	34.3	27.8	18.2	49,492	22.8
Senior (50+)	33.5	30.4	13.0	60,033	27.7
Rurality					
Major city	35.7	24.7	22.1	139,119	64.2
Regional	38.7	27.5	24.2	73,492	33.9
Remote or very remote	43.6	33.6	26.0	3972	1.8
Socio‐Economic Indexes for Areas					
1 (Most disadvantaged)	40.8	25.7	29.3	36,419	16.8
2	38.0	26.1	25.1	40,844	18.9
3	35.5	24.6	22.4	43,484	20.1
4	35.3	25.5	20.7	46,116	21.3
5 (Least disadvantaged)	35.9	27.0	19.6	49,722	23.0
Education					
Higher education	27.8	21.2	13.7	67,179	31.0
Vocational	39.7	28.8	24.4	73,720	34.0
Year 12	43.2	23.9	33.1	37,303	17.2
Year 11 and below	42.1	30.6	27.4	38,378	17.7
Partnership status					
Partnered	33.9	25.7	18.4	149,282	68.9
Previously partnered	33.4	27.3	17.8	19,590	9.0
Never partnered	48.1	25.6	40.1	47,754	22.0
Health condition					
No	37.2	25.8	23.4	179,453	82.9
Yes	34.8	25.7	20.4	37,135	17.1
Smoking status					
Non‐smoker	31.8	22.0	18.6	151,384	81.0
Smoker	57.2	41.1	40.4	35,484	19.0
Inflation‐adjusted income quartiles					
Q1 (≤ AUS$42,948)	36.4	21.1	26.5	54,161	25.0
Q2 (AUS$42,949–AUS$69,949)	36.7	24.4	24.4	54,161	25.0
Q3 (AUS$69,950–AUS$105,008)	36.1	26.2	22.0	54,159	25.0
Q4 (> AUS$105,000)	38.0	30.9	19.3	54,160	25.0
Shift work					
Normal schedule	36.5	26.2	22.2	165,991	76.6
Shift worker	37.9	24.3	25.4	50,650	23.4
Work commitment					
Full‐time	37.2	25.3	24.5	78,513	36.3
More than full‐time	41.6	31.8	24.2	70,238	32.5
Part‐time	31.3	20.1	19.7	67,445	31.2
Occupation					
Professionals	28.2	21.2	14.4	54,247	25.1
Managers	37.5	30.0	18.3	30,963	14.3
Technicians and trades workers	49.2	36.1	33.5	28,958	13.4
Community and personal service workers	35.9	20.3	25.5	23,974	11.1
Clerical and administrative workers	29.8	20.1	17.6	30,501	14.1
Sales workers	37.8	20.5	28.4	16,088	7.4
Machinery operators and drivers	50.0	35.6	35.1	13,180	6.1
Labourers	47.2	31.8	34.1	18,593	8.6
Prefer to work					
About the same	37.4	26.0	23.6	129,570	59.9
Fewer hours	34.0	26.4	17.7	55,711	25.7
More hours	40.1	23.7	30.1	31,145	14.4
Job satisfaction					
Unsatisfied or indifferent	38.9	26.2	26.1	20,283	9.4
Satisfied	36.6	25.8	22.6	196,222	90.6
Total	36.8	25.8	22.9	216,641	100.0

Table 2 provides descriptive statistics for occupation‐related variables, stratified by whether participants engaged in high‐risk alcohol consumption (any, across‐the‐week and single‐occasion). The job demands and complexity, job security and job control scales showed modest variation across high‐risk alcohol consumption categories. Further multivariable modelling (presented subsequently) will clarify the significance of these patterns.

**TABLE 2:** Mean (*M*) and standard deviation (SD) of key variables, stratified by high‐risk alcohol consumption (any, across‐the‐week or single‐occasion).

	Any		Across‐the‐week		Single‐occasion	
	Within	Exceed	Within	Exceed	Within	Exceed
	*M* (SD)	*M* (SD)	*M* (SD)	*M* (SD)	*M* (SD)	*M* (SD)
Job Demands and Complexity Scale	4.01 (1.15)	3.93 (1.13)	3.98 (1.16)	3.97 (1.12)	4.01 (1.15)	3.86 (1.14)
Job Security Scale	4.26 (1.59)	4.26 (1.58)	4.21 (1.59)	4.40 (1.58)	4.31 (1.59)	4.09 (1.57)
Job Control Scale	5.19 (1.11)	5.14 (1.12)	5.18 (1.11)	5.14 (1.12)	5.19 (1.11)	5.12 (1.11)

### 3.2 Models Examining Alcohol Consumption

Table 3 summarises the three GLMMs, which examined the occupational, sociodemographic and health correlates of high‐risk drinking. Female workers exhibited significantly reduced odds of being high‐risk drinkers (odds ratio [OR] 0.11, 95% confidence interval [CI] [0.10, 0.13]). Junior workers (aged 18–29) exhibited a twofold increased odds of any high‐risk drinking (OR 1.99, 95% CI [1.77, 2.23]) and an eightfold increased odds of single‐occasion high‐risk drinking (OR 8.07, 95% CI [7.16, 9.10]) but demonstrated reduced odds of across‐the‐week high‐risk drinking (OR 0.41, 95% CI [0.35, 0.47]) compared to senior workers (≥ 50). Workers who resided in remote areas had higher odds of all high‐risk drinking categories (ORs 1.34–1.72) compared to those living in metro areas, while regional workers demonstrated higher odds of any (OR 1.14, 95% CI [1.05, 1.24]) and across‐the‐week (OR 1.23, 95% CI [1.11, 1.35]) high‐risk drinking. Generally, more advantaged workers exhibited heightened odds of any and across‐the‐week high‐risk drinking and reduced odds of single‐occasion high‐risk drinking.

**TABLE 3:** Generalised linear mixed effects models (predicting high‐risk alcohol consumption [any, across‐the‐week or single‐occasion]).

	Any	Across‐the‐week	Single‐occasion
	OR (95% CI)	OR (95% CI)	OR (95% CI)
Sex (ref: Male)			
Female	0.11 (0.10, 0.13)***	0.08 (0.07, 0.09)***	0.16 (0.15, 0.18)***
Workforce age (ref: Senior [50+ years])			
Junior (18–29 years)	1.99 (1.77, 2.23)***	0.41 (0.35, 0.47)***	8.07 (7.16, 9.10)***
Junior‐mid (30–39 years)	1.07 (0.97, 1.19)	0.42 (0.37, 0.47)***	3.04 (2.73, 3.39)***
Mid‐senior (40–49 years)	1.04 (0.96, 1.13)	0.69 (0.63, 0.76)***	1.81 (1.65, 1.98)***
Rurality (ref: Major city)			
Regional	1.14 (1.05, 1.24)***	1.23 (1.11, 1.35)***	1.03 (0.95, 1.12)
Remote/very remote	1.40 (1.10, 1.79)**	1.72 (1.29, 2.29)***	1.34 (1.05, 1.73)*
SEIFA (ref: 1—Most disadvantaged)			
2	0.98 (0.89, 1.07)	1.15 (1.03, 1.29)*	0.91 (0.83, 1.00)*
3	0.98 (0.89, 1.08)	1.19 (1.06, 1.34)**	0.84 (0.76, 0.93)***
4	1.04 (0.94, 1.15)	1.30 (1.15, 1.47)***	0.83 (0.75, 0.92)***
5—Least disadvantaged	1.18 (1.06, 1.32)**	1.65 (1.44, 1.88)***	0.84 (0.75, 0.94)**
Education (ref: Vocational)			
Higher education	0.48 (0.43, 0.54)***	0.71 (0.61, 0.82)***	0.42 (0.38, 0.47)***
Year 12	1.14 (1.02, 1.28)*	0.89 (0.78, 1.02)	1.25 (1.12, 1.40)***
Year 11 and below	1.23 (1.09, 1.40)***	1.09 (0.94, 1.28)	1.37 (1.21, 1.55)***
Partnership status (ref: Partnered)			
Previously partnered	1.18 (1.05, 1.33)**	1.14 (0.99, 1.30)	1.28 (1.13, 1.45)***
Never partnered	2.72 (2.51, 2.94)***	1.76 (1.60, 1.93)***	3.42 (3.16, 3.70)***
Health condition (ref. No)			
Yes	0.94 (0.88, 1.00)*	0.95 (0.88, 1.02)	0.95 (0.89, 1.02)
Smoking status (ref. Non‐smoker)			
Smoker	2.80 (2.60, 3.02)***	2.91 (2.67, 3.17)***	2.57 (2.39, 2.77)***
Income quartiles (ref: Q1 [Lowest])			
Q2	1.02 (0.96, 1.09)	1.06 (0.99, 1.15)	0.98 (0.91, 1.05)
Q3	1.05 (0.97, 1.13)	1.14 (1.05, 1.24)**	0.96 (0.88, 1.03)
Q4 (Highest)	1.16 (1.07, 1.27)***	1.28 (1.15, 1.41)***	0.97 (0.89, 1.07)
Work schedule (ref. Normal schedule)			
Shift work	1.04 (0.98, 1.10)	0.94 (0.88, 1.01)	1.09 (1.02, 1.16)**
Full‐time or more hours (ref. Full‐time)			
More than full‐time	1.05 (0.99, 1.11)	1.12 (1.05, 1.19)***	0.99 (0.93, 1.05)
Part‐time	0.84 (0.78, 0.89)***	0.91 (0.84, 0.98)*	0.78 (0.73, 0.84)***
Occupation (ref. Professionals)			
Managers	1.19 (1.09, 1.30)***	1.18 (1.06, 1.30)***	1.08 (0.98, 1.19)
Technicians and trades workers	1.33 (1.20, 1.48)***	1.42 (1.26, 1.61)***	1.25 (1.12, 1.40)***
Community and personal service workers	1.37 (1.23, 1.52)***	1.10 (0.97, 1.26)	1.49 (1.33, 1.67)***
Clerical and administrative workers	1.12 (1.02, 1.23)*	1.11 (0.99, 1.24)	1.10 (0.99, 1.22)
Sales workers	1.34 (1.20, 1.49)***	1.11 (0.97, 1.27)	1.46 (1.30, 1.65)***
Machinery operators and drivers	1.32 (1.15, 1.51)***	1.24 (1.06, 1.46)**	1.32 (1.15, 1.52)***
Labourers	1.45 (1.28, 1.63)***	1.44 (1.25, 1.65)***	1.40 (1.24, 1.58)***
Preferred working hours (ref: About the same)			
Less	0.87 (0.82, 0.91)***	0.91 (0.85, 0.96)***	0.84 (0.80, 0.89)***
More	1.11 (1.04, 1.18)**	1.04 (0.96, 1.12)	1.16 (1.09, 1.24)***
Job Demands and Complexity Scale			
—	1.01 (0.98, 1.03)	1.02 (0.99, 1.05)	0.99 (0.97, 1.02)
Job Control Scale			
—	1.01 (0.99, 1.03)	1.03 (1.01, 1.05)*	0.98 (0.97, 1.00)
Job Security Scale			
—	0.98 (0.96, 1.01)	0.98 (0.95, 1.00)	0.99 (0.97, 1.01)
Job satisfaction (ref. Unsatisfied or indifferent)			
Satisfied	0.95 (0.89, 1.02)	0.93 (0.86, 1.02)	0.97 (0.90, 1.05)
Participants	21,667	21,660	21,680
Observations	155,101	154,936	155,557
ICC	0.76	0.85	0.73
Marginal *R* ^2^/conditional *R* ^2^	0.15/0.80	0.10/0.87	0.20/0.79

Abbreviations: CI, confidence intervals; ICC, intra‐class correlation; OR, odds ratio; SEIFA, Socio‐Economic Indexes for Areas.

*** *p* = 0.001.

** *p* = 0.01.

* *p* = 0.05.

Compared to workers with vocational education, participants with a higher education degree had significantly lower odds of all the high‐risk drinking categories (ORs 0.42–0.71). Conversely, workers whose highest educational attainment was Year 12 or Year 11 or less were associated with increased odds of any (ORs 1.14 and 1.23) and single‐occasion (ORs 1.25 and 1.37) high‐risk drinking, compared to those with vocational education. Individuals who had never been partnered showed significantly higher odds of exceeding all high‐risk drinking categories (ORs 1.76–3.42) compared to those who are currently partnered. Having a health condition was associated with slightly lower odds of any high‐risk drinking (OR 0.94, 95% CI [0.88, 1.00]). Smoking was the modifiable risk factor associated with the highest odds of any (OR 2.80, 95% CI [2.60, 3.02]), across‐the‐week (OR 2.91, 95% CI [2.67, 3.17]) and single‐occasion (OR 2.57, 95% CI [2.39, 2.77]) high‐risk drinking. Higher income quartiles were associated with increased odds of any and across‐the‐week high‐risk drinking relative to the lowest quartile.

Regarding occupation‐related variables, shift workers were associated with increased odds of single‐occasion high‐risk drinking (OR 1.09, 95% CI [1.02, 1.16]). Individuals who worked more than full‐time were associated with increased odds of high‐risk drinking across the week (OR 1.12, 95% CI [1.05, 1.19]), while those who worked part‐time displayed decreased odds of all high‐risk drinking categories (ORs 0.78–0.91) compared to full‐time workers. Compared to professionals, all occupation types showed increased odds for at least one category of high‐risk drinking, with labourers consistently exhibiting the highest—or among the highest—odds for all categories (ORs 1.40–1.45). Compared to workers who were content with their working hours, workers who preferred to work more hours showed increased odds of any (OR 1.11, 95% CI [1.04, 1.18]) and single‐occasion high‐risk drinking (OR 1.16, 95% CI [1.09, 1.24]), whereas those who preferred to work fewer hours were associated with lower odds of all high‐risk drinking categories (ORs 0.84–0.91). Perceived job demands and complexity, as well as perceived job security and job satisfaction, were not associated with high‐risk alcohol consumption. Higher perceived job control was associated with a slight increase in the odds of across‐the‐week high‐risk drinking (OR 1.03, 95% CI [1.01, 1.05]).

## 4 Discussion

This study investigated the extent to which occupational factors explain the variance in high‐risk alcohol consumption among Australian workers, beyond what is explained by sociodemographic and health variables. When compared to those working full‐time hours, individuals working more than full‐time hours were associated with high‐risk drinking across the week, whereas those working part‐time showed reduced odds of all high‐risk drinking categories. Shift workers exhibited increased odds of single‐occasion high‐risk alcohol consumption. Compared to professionals, all occupation types showed increased odds in at least one of the high‐risk drinking categories, with labourers consistently being among those with the strongest associations. Individuals who preferred to work more hours exhibited greater odds of any and single‐occasion high‐risk drinking, whereas those who preferred to work fewer hours had reduced odds of all high‐risk drinking categories. Contrary to previous research [27, 28, 29], perceived job demands and complexity—commonly referred to as job stress—and job security were not associated with high‐risk drinking. Higher perceived job control demonstrated a weak positive association with high‐risk drinking across the week. Job satisfaction was not associated with high‐risk alcohol consumption—an unexpected finding [9, 26]. These findings suggest that factors internal to the workplace, as proposed by Frone and Bamberger [10], explain some of the variance in high‐risk alcohol consumption. However, several factors external to the workplace (i.e., sociodemographic and health variables) explained much more of the variance in high‐risk alcohol consumption.

### 4.1 Occupational Factors and High‐Risk Alcohol Consumption

Shift workers were no more likely to engage in high‐risk drinking in general, and across the typical week, than those who worked regular schedules. However, they were more likely to be single‐occasion high‐risk drinkers. This result broadly supports previous research, which has found that shift workers exhibited increased odds of engaging in single‐occasion high‐risk alcohol consumption but not high‐risk drinking across the week [18, 22, 23]. Shift workers may be less likely to consume alcohol on a regular basis, as their shifts may conclude at times when alcohol is not commonly consumed (e.g., 7 AM), thereby limiting the opportunities for them to consume alcohol. Yet, they are more likely to engage in single‐occasion high‐risk alcohol consumption, indicating that they may be making up for their fewer opportunities by drinking more on the days that they do consume alcohol [17]. Moreover, shift workers often maintain the misconception that alcohol aids in falling asleep [17, 20]; therefore, it is essential for employers to actively dispel this myth and educate employees that alcohol has the opposite effect [21, 22].

The present study found that those working more than 40 h had increased odds of high‐risk drinking across the week compared to full‐time workers—a finding that supports previous research [15, 24, 25]. This could suggest that prolonged work hours lead to heightened stress levels, which tend to accumulate as hours increase, potentially resulting in a greater likelihood of workers resorting to alcohol consumption as a coping mechanism. However, this is merely speculation, as our analytic technique precludes us from drawing causal inferences. Preferring to work more hours was associated with increased odds of any and single‐occasion high‐risk alcohol consumption—consistent with Dorrian and Skinner [18]—which may be reflective of underlying traits (e.g., financial strain, workaholism) that may predispose individuals to high‐risk drinking. Workplace interventions that include education about the potential harms excessive work hours may engender, as well as stress management resources and policies promoting work–life balance, may be effective in lessening the relationship between extended work hours and high‐risk alcohol consumption.

### 4.2 Sociodemographic and Health Factors Related to High‐Risk Alcohol Consumption

To address the broader context through which occupational factors influence drinking patterns, this investigation also examined sociodemographic and health‐related variables, which are referred to as factors external to the workplace in the Frone and Bamberger [10] model. Female workers were observed to have an 89% decrease in the odds of any high‐risk drinking compared to male workers—a finding corroborated by population prevalence data [7]. Age was associated with high‐risk drinking, whereby the junior workforce (18–29 years of age) was more likely to drink at any and single‐occasion high‐risk levels, while the senior workforce (50+) was more likely to drink at high‐risk levels across the week, which is consistent with past research [15]. Workers who smoked exhibited an almost threefold increase in the odds of being a high‐risk drinker compared to those who did not—consistent with findings from previous research [15, 49]. Since co‐use of tobacco and alcohol is linked to greater long‐term health risks [2, 50, 51], there is a need for education strategies and workplace screening and brief interventions to assess both smoking and alcohol use together rather than in isolation.

### 4.3 Limitations and Directions for Future Research

The authors emphasise that the analyses demonstrate associations between alcohol consumption and predictor variables rather than causal relationships. Furthermore, due to the unbalanced survey design, survey weights could not be applied; consequently, the analyses—including prevalence rates—are not representative of the Australian population. The findings of this study inform several avenues for future research. It was found that shift work had a mixed relationship with alcohol consumption. Future research might benefit from a more granular examination of how different shift work rosters (e.g., night, split, rotating shifts) affect alcohol consumption. This information is particularly relevant for industries where the impairment resulting from alcohol may have especially severe consequences, such as health care and transport. Furthermore, it would be beneficial to understand in which contexts workers drink alcohol, as well as their drinking motives—such as whether they report using it to cope, as a sleeping aid or as a social lubricant. The existing literature may gain from comparing high‐risk alcohol consumption patterns between workers in safety‐sensitive industries and those in non‐safety‐sensitive industries. Future studies may also consider examining the association between the variables considered in the present study and the use of illicit substances and tobacco. For instance, substances such as cannabis may be used by individuals facing significant work‐related stress, long hours and shift work to relax and aid sleep.

## 5 Conclusions

Occupational factors—including full‐time employment, shift work and dissatisfaction with work hours—are significantly associated with high‐risk alcohol consumption among employed Australians, even after controlling for sociodemographic and health variables. Individuals working excessive hours or desiring to work more hours, may be particularly vulnerable, which suggests that workplaces where individuals tend to have a culture of working longer hours may benefit most from health promotion interventions aimed at reducing high‐risk alcohol consumption. Furthermore, shift work was associated with single‐occasion high‐risk alcohol consumption, highlighting the necessity for workplaces to raise awareness regarding the harms of high‐risk single‐occasion drinking and to dispel myths surrounding alcohol as an effective sleep aid. The observation that age and sex were strongly associated with high‐risk alcohol consumption indicates that tailored strategies may be necessary to address harmful alcohol use in the workforce. Moreover, the association between smoking and high‐risk alcohol consumption reinforces the need for universal, workplace‐based interventions addressing substance use more generally.

## Author Contributions

Each author certifies that their contribution to this work meets the standards of the International Committee of Medical Journal Editors.

## Funding

This work was supported by the Australian Government Department of Health, Disability and Ageing.

## Ethics Statement

The authors have nothing to report.

## Conflicts of Interest

The authors declare no conflicts of interest.

## Data Availability

The data underlying this article were provided by the Australian Government Department of Social Services Longitudinal Studies Dataverse. Data can be accessed on request at: https://doi.org/10.26193/NBTNMV.
